# 90 The association between risk of depression and combined vigorous physical activity and screen time behaviours in French-speaking Belgian adolescents

**DOI:** 10.1093/eurpub/ckae114.186

**Published:** 2024-09-26

**Authors:** Wassila Assakali, Katia Castetbon, Morgane Eggen, Emma Holmberg, Caroline Mertens

**Affiliations:** Service d’Information, Promotion, Education Santé (SIPES), Research Centre in “Epidemiology, Biostatistics and Clinical Research”, School of Public Health, Université libre de Bruxelles (ULB), Brussels, Belgium; Service d’Information, Promotion, Education Santé (SIPES), Research Centre in “Epidemiology, Biostatistics and Clinical Research”, School of Public Health, Université libre de Bruxelles (ULB), Brussels, Belgium; Service d’Information, Promotion, Education Santé (SIPES), Research Centre in “Epidemiology, Biostatistics and Clinical Research”, School of Public Health, Université libre de Bruxelles (ULB), Brussels, Belgium; Service d’Information, Promotion, Education Santé (SIPES), Research Centre in “Epidemiology, Biostatistics and Clinical Research”, School of Public Health, Université libre de Bruxelles (ULB), Brussels, Belgium; Service d’Information, Promotion, Education Santé (SIPES), Research Centre in “Epidemiology, Biostatistics and Clinical Research”, School of Public Health, Université libre de Bruxelles (ULB), Brussels, Belgium

## Abstract

**Purpose:**

While there is a clear consensus on the positive effect of physical activity on reducing the risk of depression, the literature on the association between screen time and risk of depression is rather conflicting. Also, no clear consensus has been reached on whether being sufficiently active could alleviate the negative effects of being sedentary on mental health. The purpose of this study was to investigate the association of combined vigorous physical activity (VPA) and screen time (ST) behaviours with the risk of depression (RD) in Belgian adolescents.

**Methods:**

The analysis used data collected through self-reported questionnaires from 8412 adolescents aged 10 to 20 included in the 2022 “Health Behaviour in School-aged Children” (HBSC) study in French-speaking schools. RD was measured using the World Health Organisation Five Well-being Index (WHO-5), with scores ≤28/100 indicating RD. VPA and ST behaviours were identified by combining data on ST (TV, video games and internet use) and VPA in three categories: (1) low ST (total weekly ST < 4hours/day), independently of VPA; (2) high VPA-high ST (VPA≥3 times/week and total weekly ST ≥ 4hours/day) and (3) low VPA-high ST (VPA<3 times/week and total weekly ST ≥ 4hours/day). The association between RD and VPA-ST behaviours was measured using adjusted binary logistic regression.

**Results:**

In 2022, 15.4% had low ST, 40.6% high VPA-high ST, and 44.0% low VPA-high ST behaviours. Moreover, 13.2% were at risk of depression. This prevalence was eight times higher in low VPA-high ST (62.9%) and four times higher in high VPA-high ST (29.5%) than in low ST (7.6%) adolescents. After adjusting for sociodemographic characteristics, body mass index and sleep duration, adolescents with low VPA-high ST behaviours were more likely to be at risk of depression (aOR=1.17, 95%CI: 1.31-2.35) compared to those with low ST, but this was not the case for those who had high VPA-high ST (aOR=1.18, 95%CI: 0.81-1.71).

**Conclusion:**

These findings suggest that having either low ST or sufficient VPA may be protective for RD in adolescents. With ST increasing and PA remaining low in French-speaking Belgian adolescents, health promotion initiatives should continue to promote both PA and the reduction of sedentary behaviours in adolescents.

